# Bactericidal activity and biocompatibility of ceragenin-coated magnetic nanoparticles

**DOI:** 10.1186/s12951-015-0093-5

**Published:** 2015-05-01

**Authors:** Katarzyna Niemirowicz, Urszula Surel, Agnieszka Z Wilczewska, Joanna Mystkowska, Ewelina Piktel, Xiaobo Gu, Zbigniew Namiot, Alina Kułakowska, Paul B Savage, Robert Bucki

**Affiliations:** Department of Microbiological and Nanobiomedical Engineering, Medical University of Bialystok, Mickiewicza 2c, 15-222 Bialystok, Poland; Institute of Chemistry, University of Bialystok, 1 Hurtowa, 15-399 Bialystok, Poland; Department of Materials and Biomedical Engineering, Białystok University of Technology, 15-351 Białystok, Poland; Department of Physiology, Medical University of Białystok, 15-230 Białystok, Poland; Department of Neurology, Medical University of Bialystok, 15-230 Bialystok, Poland; Department of Chemistry and Biochemistry, Brigham Young University, Provo, UT USA; Department of Physiology, Pathophysiology and Microbiology of Infections, The Faculty of Health Sciences of the Jan Kochanowski University in Kielce, 25-317 Kielce, Poland

**Keywords:** Antibacterial activity, *Pseudomonas aeruginosa*, Ceragenins, Magnetic nanoparticles

## Abstract

**Background:**

Ceragenins, synthetic mimics of endogenous antibacterial peptides, are promising candidate antimicrobial agents. However, in some settings their strong bactericidal activity is associated with toxicity towards host cells. To modulate ceragenin CSA-13 antibacterial activity and biocompatibility, CSA-13-coated magnetic nanoparticles (MNP-CSA-13) were synthesized. Transmission electron microscopy (TEM), Fourier transform infrared spectroscopy (FT-IR), differential scanning calorimetry (DSC) and thermogravimetric analysis (TGA) were used to characterize MNP-CSA-13 physicochemical properties. Bactericidal action and ability of these new compounds to prevent *Pseudomonas. aeruginosa* biofilm formation were assessed using a bacteria killing assay and crystal violet staining, respectively. Release of hemoglobin from human red blood cells was measured to evaluate MNP-CSA-13 hemolytic activity. In addition, we used surface activity measurements to monitor CSA-13 release from the MNP shell. Zeta potentials of *P. aeruginosa* cells and MNP-CSA-13 were determined to assess the interactions between the bacteria and nanoparticles. Morphology of *P. aeruginosa* subjected to MNP-CSA-13 treatment was evaluated using atomic force microscopy (AFM) to determine structural changes indicative of bactericidal activity.

**Results:**

Our studies revealed that the MNP-CSA-13 nanosystem is stable and may be used as a pH control system to release CSA-13. MNP-CSA-13 exhibits strong antibacterial activity, and the ability to prevent bacteria biofilm formation in different body fluids. Additionally, a significant decrease in CSA-13 hemolytic activity was observed when the molecule was immobilized on the nanoparticle surface.

**Conclusion:**

Our results demonstrate that CSA-13 retains bactericidal activity when immobilized on a MNP while biocompatibility increases when CSA-13 is covalently attached to the nanoparticle.

## Background

Ceragenin CSA-13 belongs to family of synthetic mimics of cationic antimicrobial peptides (CAPs) that display strong antibacterial activities [1,2]. Cationic lipids such as CSA-13 are easier to prepare and purify than antimicrobial peptides [3]. They are also resistant to digestion by proteases that are commonly present at infection sites [4]. Numerous reports describe CSA-13 activity against multidrug-resistant bacteria, lipid-enveloped viruses and parasites [3,5,6]. The mechanism of CSA-13 action involves direct interaction with negatively charged bacterial membrane molecules including lipopolysaccharide and phosphatidylglycerol, which results in membrane permeabilization [7,8]. However, CSA-13′s ability to compromise lipid organization in bacterial membranes is not completely selective, and, at higher concentrations, CSA-13 can affect the lipid organization within host cell membranes, which in the case of RBCs leads to hemolysis [7,8]. Methods intended to reduce potential harmful effects of CSA-13 and other ceragenins are provided by nanotechnology strategies and the use of nanoparticles as drug delivery systems (DDS) [9,10]. Generally, this approach may prove beneficial in providing a larger therapeutic window (i.e., the difference in effective *vs.* toxic concentrations). Among many types of nanoparticles, magnetic nanoparticles (MNPs) are one of the most promising materials in nanomedicine [11]. They can be delivered to a specific area via a magnetic field [12-14]. Targeting provided by nanoscale-based drug delivery limits and helps control drug toxicity. Additionally, MNP surface functionalization provides the opportunity to attach different active molecules such as drugs or homing ligands and combine diagnostic and therapeutic activities within the same structure [15]. In this study, we developed a novel magnetic nanosystem made out of an iron oxide core, aminosilane layer and CSA-13. Our results suggest that immobilization of CSA-13 on the MNP surface significantly reduces membrane toxicity to RBCs and increases antimicrobial activity against selected bacteria.

## Results and discussion

**Figure 1:**
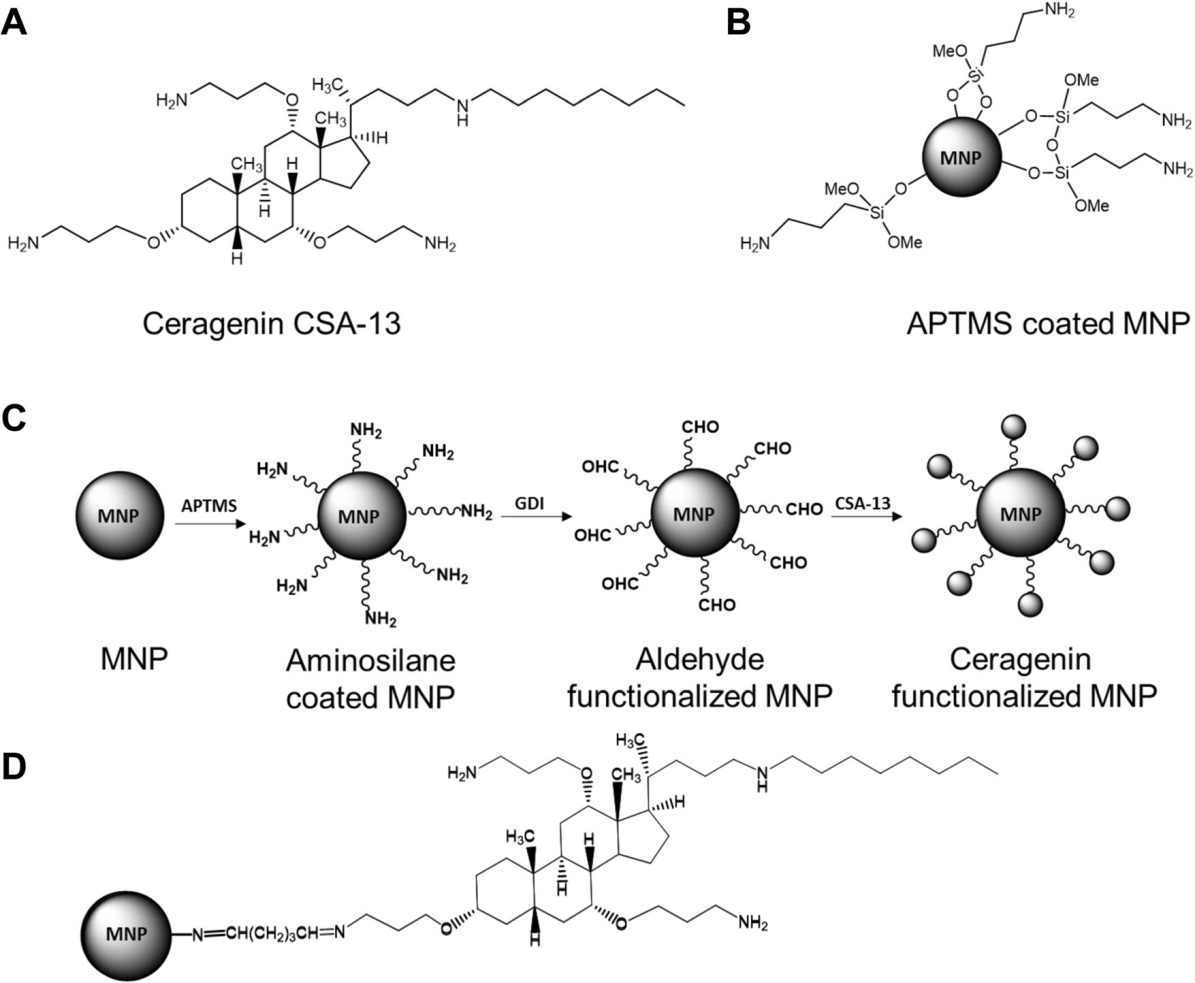
Schematic representation of MNP-CSA-13 synthesis. The structures of ceragenin CSA-13 (panel **A**) and APTMS coated magnetic nanoparticles (panel **B**). The overall surface modification procedures: In the first step bare MNPs were coated with an aminosilane layer (APTMS), then aminosilane-coated MNPs were reacted with gluteraldehyde, a process resulting in the formation of terminal aldehyde groups able to interact with ceragenin CSA-13 through imine bond formation (panel **C**). Final structure of MNPs functionalized with CSA-13 (panel **D**).

**Figure 2:**
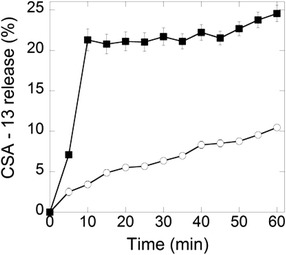
pH-dependent release of CSA-13 from MNP-CSA-13. CSA-13 release (hydrolysis of imine bond) was monitored by recording changes of surface tension at an air-water interface of a MNP-CSA-13 suspension. A rapid increase of CSA-13 release during the first 10 minutes after placing MNP-CSA-13 in a low pH buffer (pH = 5) was observed (black squares). After this time the shape of the curves indicates further gradual release. On the other hand, at pH = 7.4, slow, but progressive release of CSA-13 molecules from MNP-CSA-13 was observed (white circles). Data represent mean ± SD from 3 experiments.

The synthesis of MNP-CSA-13 and the surface modification process is shown in Figure 1. The MNP magnetic core was obtained following a modification of Massart’s method [16]. After formation, magnetic nanoparticles were treated with (3-aminopropyl)trimethoxysilane (APTMS) to link the amino-terminated silica to their surface. Then amine functionalized silica nanospheres were treated with glutaraldehyde, to obtain a platform for CSA-13 immobilization. The terminal aldehyde groups from this surface are able to react with the primary amine groups of CSA-13. This reaction results in an imine bond between the surface and CSA-13. We anticipate that amine group at the C3 position in CSA-13 is the primary site of reactivity because it is less sterically hindered than other amines in the molecule. Imine formation for CSA-13 immobilization was selected because treatment of imines with an excess of water leads to their hydrolysis back to an aldehyde and an amine, especially in the presence of acid. During infection and inflammation a decrease of the pH frequently occurs [17,18]. Therefore, MNP-CSA-13 could be used as carriers for controlled antibiotic delivery to kill microorganisms at infection sites where the pH is usually less than 6 [19,20]. The release of CSA-13 from MNP-CSA-13, subjected to a magnetic field, was assessed by measuring the increase of surface tension. As CSA-13 is released the surface tension is altered (Figure 2). The release rate was expected to depend on local pH, and over a one hour a much slower release rate was observed at pH 7.4 (10% release) as compared to pH 5 (25% release). We did not observe differences between intrinsic surface activity of CSA-13 at pH 7.4 and 5 (data not shown). We conclude that lower pH accelerates imine bond hydrolysis, liberating CSA-13 from the nanoparticle. It is worth noting that rapid CSA-13 release during the first 20 minutes is potentially due to a burst effect [21]. The ability to control CSA-13 release from nanoparticles in a low pH indicates a potential benefit of using this nanosystem in eradication of *Helicobacter pylori*. High susceptibility of *H. pylori* to CSA-13 was previously described [22]. Moreover, the observed burst effect of CSA-13 release may be useful for certain applications, especially for wound treatment, targeted delivery and pulsatile release [23].

**Figure 3:**
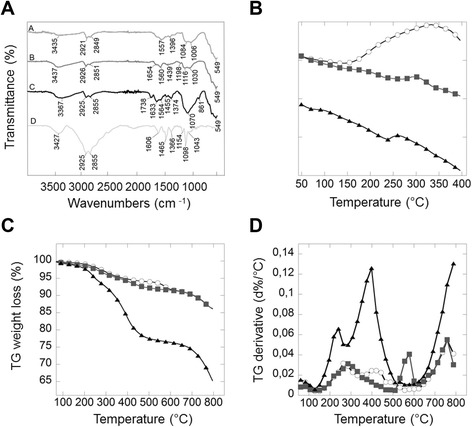
Physicochemical properties of synthesized magnetic nanoparticles. ATR FT-IR spectra of the aminosilane- (curve A), glutaraldehyde - (curve B), CSA-13 (curve C) functionalized MNPs and free CSA-13 (curve D) (panel **A**). Panels **B-D** show thermal properties of aminosilane- (light gray circle line), glutaraldehyde - (square gray line) and CSA-13 (triangle black line) functionalized MNPs. Differential scanning calorymetry analysis curves, thermogravimetric analysis curves and differential thermal analysis represent panels **B, C** and **D** respectively. Data from one experiment performed in triplicate are shown.

Figure 3A shows FT-IR spectra (A – C) for aminosilane-, glutaraldehyde- and ceragenin-functionalized magnetic nanoparticles (MNP@NH_2_; MNP@NH=CH(CH_2_)_3_CHO; MNP-CSA-13 respectively). The curve D represented FT-IR spectrum of unbound CSA-13. The existence of a magnetic core in all samples is indicated by a band at ~550 cm^−1^, which corresponds to the Fe-O stretching mode of Fe_3_O_4_. The spectrum of MNP@NH_2_ shows a broad band at ~ 1000–1090 cm^−1^ associated with Si-O, Si-O-Si and Fe-O-Si stretching vibrations. In all samples, the bands around 1557 cm^−1^ and above 3300 cm^−1^ correspond to N-H bending and stretching vibrations, respectively. After functionalization with glutaraldehyde the stretching vibration of the C = O bond around 1654 cm^−1^ was recorded. CSA-13 attachment to the MNP surface was detected by a band at 1738 cm^−1^, which corresponds to the imide stretching band. Additionally, the imide in-plane stretching bands were found at 1564 cm^−1^. The presence of C-H stretching modes around 2930 cm^−1^ and 2850 cm^−1^ associated with implemented ethyl and alkyl groups further confirm the presence of a silica shell on the MNP surface and CSA-13 functionalization. The characteristic band for free CSA-13 was present within MNP-CSA-13, indicating that CSA-13 molecules were immobilized on the nanocomposite surface.

The thermal properties of MNP-CSA-13 were characterized by differential scanning calorimetry (DSC) (Figure 3B). The heating curves of aminosilane- and glutaraldehyde- and ceragenin-functionalized MNPs indicates differences in the chemical nature of coating. At temperatures higher than 150°C the breakdown of the organic skeleton (decomposition of methoxyl and propylamine groups) was observed in the DSC curve of MNP@NH_2_ (empty circles). Analysis of the curves for glutaraldehyde functionalized MNPs (gray squares) and CSA-coated MNP (black triangles) indicate that these peaks can’t be distinguished, which can be explained by the functionalization of the aminosilane surface by glutaraldehyde and ceragenin respectively. In the curve for glutaraldehyde- and CSA-13-functionalized MNPs, a large endothermic transition was found from 150 to 400°C. Additionally, a high endothermic peak for MNP-CSA-13 at 235°C was observed. Figure 3 panels C and D show the thermogravimetric analysis results for the aminosilane-, glutaraldehyde-, and CSA-13- functionalized magnetic nanoparticles, respectively. Bare MNP shows 6% weight loss in the temperature range 50–800°C, which is directly associated with the removal of adsorbed water and decomposition of hydroxyl surface groups [24]. The thermogravimetric analysis curves (Figure 3C) show that the weight loss for aminosilane- and glutaraldehyde-coated MNPs during the performed analysis is about 8%. Curve comparison of glutaraldehyde modified MNPs and aminosilane modified MNPs, (Figure 3D), revealed a characteristic peak at 570°C. After glutaraldehyde decomposition, the shape of both curves did not differ. The thermogravimetric analysis of CSA-13 functionalized MNPs (Figure 3C) showed a total weight loss of 35%. The 14% weight loss of the MNP-CSA-13 (compared to the weight loss of bare and MNP@NH_2_) indicated successful CSA-13 immobilization on the MNP surface, with ~ 14% CSA-13 content. For MNP-CSA-13, we observed two decomposition peaks, with the first at 240°C and the second at 400°C (Figure 3D). A second peak indicated decomposition of the aminosilane layer, which was recorded for all analyzed samples. Based on weight lost and total amount of amine group on the MNP surface, we calculated that the number of CSA-13 molecules was 3.53 × 10^13^, which corresponds to a number of molecules 20 times lower per μg present than in the original volume of CSA-13 solution used for MNP functionalization.

**Figure 4:**
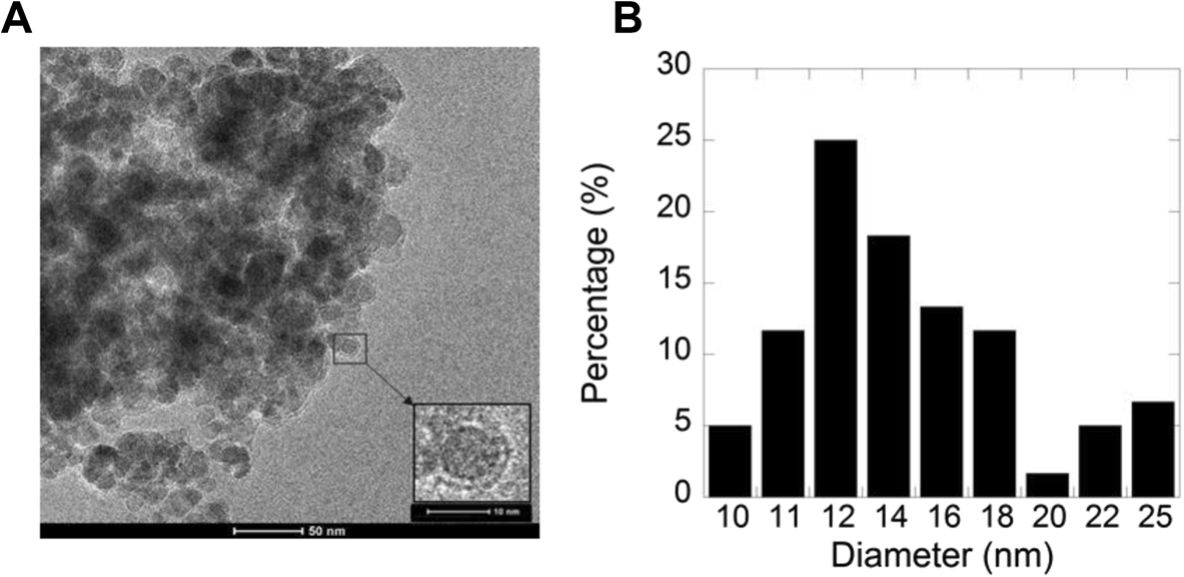
Transmission electron microscopy (TEM) images of MNP-CSA-13. Transmission electron microscopy images (pane **A**) and size distribution (panel **B**) of MNP-CSA-13. Ceragenin-loaded magnetic nanoparticles had an iron oxide core of 9 ± 2 nm. MNP functionalization with CSA-13 results in a MNP size increase. Data from one experiment performed in triplicate are shown.

A relatively narrow size distribution of magnetic nanoparticles functionalized with CSA-13 is shown in Figure 4. Particles were spherical with a diameter as determined from the TEM image of 14 nm ± 2 nm (the size was calculated using 100 randomly selected particles). Nanoparticles should be well separated due to the repulsion between CSA-13 molecules in the coating layer, but the observed MNPs clusters can be explained by the drying process in the preparation for TEM measurements. When compared to previously characterized amino- functionalized MNPs, the size of CSA-13 functionalized MNPs did not significantly differ [25]. Additionally, the accuracy of TEM size analysis was in agreement with XRD analysis (data not shown) [25].

**Figure 5:**
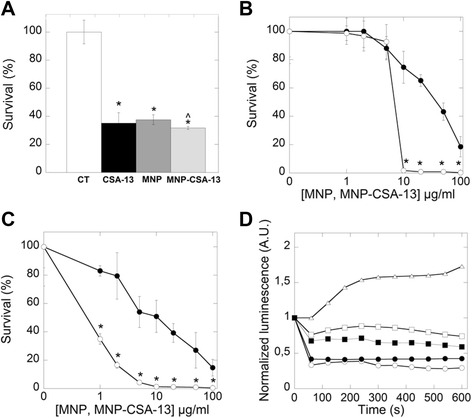
Bactericidal activity of MNP@CSA-13 against *P. aeruginosa* strains. Bactericidal activity of CSA-13 (0.7 μg/ml), MNPs (4.3 μg/ml) and MNP-CSA-13 (5 μg/ml) against *P. aeruginosa* PAO14 (CT - control) (panel **A**). To compare antibacterial potential of MNP, CSA-13 and MNP-CSA-13 against *P. aeruginosa*, CSA-13 concentration was estimated based on physicochemical data and yield of reaction. Panels **B** and **C** show survival of PAO1 and a clinical strain of *P aeruginosa* isolated from cystic fibrosis sputum in the presence of MNPs (black circles) and MNP-CSA-13 (white circles) respectively. Reduction of *P. aeruginosa* Xen 5 (white triangels) chemiluminescence signal (~10^8^ CFU/ml) after colistin – COL (100 μg/ml; white square), MNP (100 μg/ml; black square), CSA-13 (100 μg/ml; black circles) and MNP-CSA-13 (100 μg/ml; white circles) addition – panel **D**. Data represent mean ± SD, n = 3 with * statistical significance compared to untreated bacteria cells (p ≤ 0.05); ^ statistical significance compared to bacteria treated with free CSA-13 (p ≤ 0.2) (panel **A**).

*P. aeruginosa* is an opportunistic pathogen that frequently causes hospital infection, affecting mostly immunocompromised patients and those suffering from cystic fibrosis [26,27]. Using physicochemical evaluation, the total amount of CSA-13 on the MNPs surface was determined and bactericidal activity of an equal CSA-13 concentration (free CSA-13 and CSA-13 attached to MNPs) per ml were compared. Figure 5A shows that immobilization of CSA-13 on the nanoparticle surface did not affected its antibacterial activity against *P. aeruginosa*, and this observation is in agreement with a previous study where CSA-13 in combination with pluronic acid was tested [28]. Bactericidal activity against clinical isolates of *P. aeruginosa* strains shows that more than 60% of bacteria were killed using a 1 μg/ml dose, and at concentration of 10 μg/ml no bacteria growth was observed (Figure 5C). Interestingly, a dose-dependent decrease of bacteria growth was observed for uncoated MNPs, with a 80% decrease at 100 μg/ml. The decline of *P. aeruginosa* Xen 5 chemiluminescence after treatment with MNP-CSA-13 is shown in Figure 5D. Comparison of bacteria luminescence after MNP-CSA-13 addition suggests that the nanocomposite is more efficient in killing bacteria than CSA-13 alone. The decrease of chemiluminescence, which indicates the ability of MNP-CSA-13 to affect bacteria metabolism, reached up to 70%, as compared to colistin (~24%), and unfunctionalized MNPs (~40%). These data support the hypothesis that a synergistic effect of MNPs and CSA-13 might exist. Novel formulations using lipids and polymeric nanostructures for delivery of antimicrobial agents may be useful in other applications as well [29]. A magnetic drug delivery system was previously proposed for antibiotics such as chloramphenicol (successfully loaded into OA/SDS-coated MNPs) [30], nystatin [31] and streptomycin [24], both loaded on chitosan coated MNPs; however, the attaching process was achieved by chemical adsorption, based on non-specific electrostatic or hydrophobic interactions. The covalent immobilization of antibiotics proposed here possesses some advantages compared to chemical adsorption. Recent reports show that immobilization of antimicrobial peptides (AMPs) onto a biomaterial surface helps to avoid AMP limitations, such as short half-life and toxicity associated with higher concentrations of soluble peptides [32]. Additionally, Zhao et al. argued that advantages of attachment by chemical reaction also include low incidence of side effects and non-accumulation in tissues (brain, liver and spleen) [33]. Moreover, covalent bonding allows the pH-depending control of substance release, due to the mechanism of the hydrolysis reaction [34].

**Figure 6:**
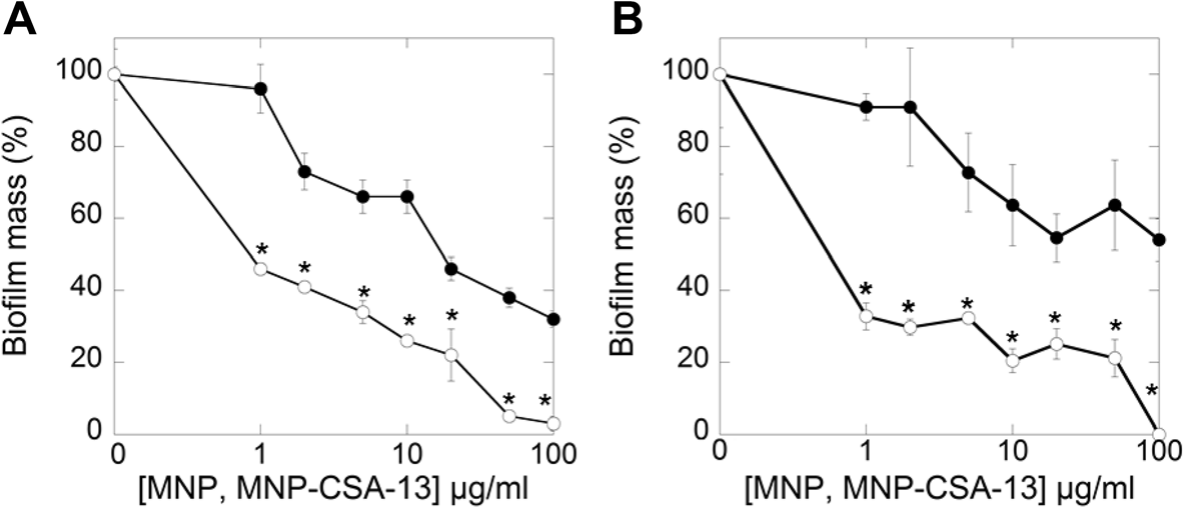
MNP-CSA-13 activity against bacteria biofilm formation. Bactericidal activity of MNPs (black circles) and MNP-CSA-13 (white circles) against biofilm forming *P. aeruginosa* PA01 (panel **A**) and *P. aeruginosa* clinical strain (panel **B**). Panels **A** and **B** represent effective killing of bacteria embedded in biofilm. Results represent mean ± SD obtained from 3 experiments. * statistical significance compared to bacteria treated with unfunctionalized MNPs.

Bacteria growth in biofilm form is associated with increased resistance to antibiotic treatment. Additionally, biofilm plays a key role in chronic *Pseudomonas* infections [35-37]. Therefore, new approaches to prevent biofilm formation are of great interest. As indicated in Figure 6 MNP-CSA-13 was able to prevent biofilm formation and effectively kill bacteria embedded into the biofilm matrix. At MNP-CSA-13 concentration of 1 μg/ml, biofilm formation decreased by ~ 40%. Total reduction of adhesion of *P. aeruginosa* on a polystyrene surface was observed at a concentration of 100 μg/ml. A similar effect was obtained for both tested *P. aeruginosa* strains (PAO1 and a selected clinical strain). Collected results are strongly supported by a recent study showing that CSA-13 is active against bacteria forming biofilms. Previous reports indicate that more than 50% of bacteria cells in such networks were killed at 50 μg/ml. Total reduction of live bacteria cells was observed with the concentration of 100 μg/ml [38]. Additionally, another study shown that CSA-13 inhibits the adhesion of *P. aeruginosa* to an abiotic surface and reduces biofilm mass [39]. Our results indicate that MNP-CSA-13 is useful in preventing bacteria biofilm formation, and it may have higher activity than previously described nanosystems [40]. Iron oxide nanoparticles coated by a carboxylate shell at a concentration of 1000 μg/ml were able to kill up to 33% of *S. aureus* after initial biofilm formation over a 24 hour time period [40]. Similarly, another study demonstrated significant reduction in biofilm growth for *S. aureus* and *P. aeruginosa* in the presence of iron oxide and gold nanoparticles at a concentration range 50–150 μg/ml [41]. Hetric *et al.* revealed that nitric-oxide releasing silica nanoparticles strongly inhibit biofilm formation by Gram-negative and Gram-positive bacteria [42]. Abdelghany *et al.* showed that gentamycin-loaded polylactide-co-glycolide nanoparticles possess excellent potential against a pre-formed *P. aeruginosa* biofilm [43].

**Figure 7:**
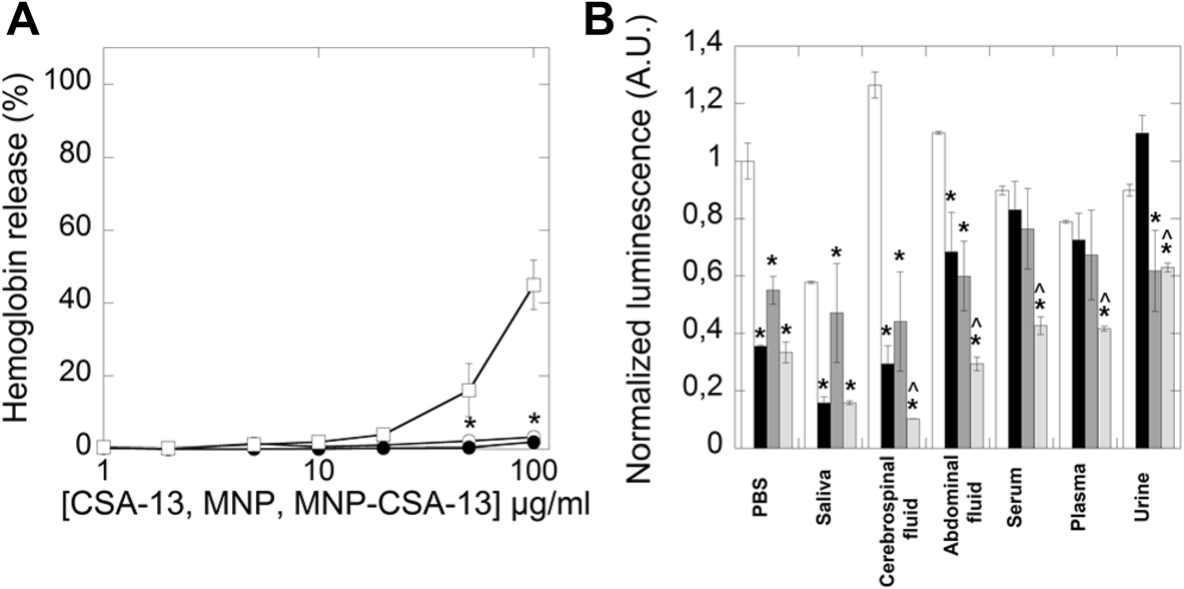
Biocompatibility and antibacterial activity of MNP-CSA-13 in different body fluids. Hemoglobin release from human red blood cells (RBCs) after CSA-13 (white squares), MNP (white circles) and MNP-CSA-13 (black circles) addition (panel **A**). Antibacterial activity of MNP-CSA-13 against *Pseudomonas aeruginosa* Xen 5 in different human body fluids assessed using chemiluminescence measurement (panel **B**). In each condition the white column indicates luminescence of control samples. The black column indicates the luminescence signal after free ceragenin CSA-13 (100 μg/ml) treatment. The gray column indicates luminescence signal after addition of MNPs (100 μg/ml). Light gray column show luminescence signal after addition of MNP-CSA-13. Results represent mean ± SD, n = 3; * statistical significance compared to untreated control, ^ compared to bacteria treated with CSA-13 (p ≤ 0.05).

The toxicity attributed to AMPs is frequently correlated to high concentrations used to compensate for their relatively short half-life due to rapid protease digestion or aggregate formation [44,45]. Ceragenins, including CSA-13, are likely cause red blood cell (RBC) hemolysis at high concentrations as a consequence of their membrane activity. This effect is caused by membrane destruction after insertion [46,47]. An understanding of the mechanisms governing magnetic nanoparticle interactions with RBC membranes is necessary to create an optimal nanosystem for medicinal applications [48]. Data in Figure 7A indicate that MNP-CSA-13 did not affect RBC membrane permeability at a concentration range of 1 – 100 μg/ml. This concentration range of the nanoparticles effectively kills planktonic *P. aeruginosa* as well as the bacteria in a mature biofilms. This result suggests that attachment of CSA-13 to the MNP surface can be used to control CSA-13 toxicity and potentially toxicity of other cationic lipids using covalent immobilization on the nanoparticle surface. Recent data show that CSA-124 (thiolated derivative of CSA-13) binding to silver nanoparticles (SNP) via non-specific interactions (thermodynamically favorable silver-sulfur bonding) caused 100 percent hemolysis at a concentration of 50 μg/ml, which is 1.5 times higher compared to CSA-124 alone [49]. Our study indicates that CSA-13, at these same concentrations, covalently attached to MNPs caused ~1% hemolysis. These data provide evidence that chemical adsorption of drugs onto nanoparticle surfaces decreases hemolysis. Thus, we show the advantage of covalent binding compared with non-specific chemical adsorption. Ruden *et al.* reported that increasing the concentration of silver ions in combination with various antimicrobial peptides enhanced antibacterial activity. However, the combination of those peptides with silver nanoparticles did not increase hemolysis [50]. Another study indicated that RBCs exposed to gold coated magnetic nanoparticles induces hemolysis rates of under 5% [51]. Similarly, encapsulation of daunorubicin on self-assembled iron oxide magnetic nanoparticles presented good hemocompatibility, with a hemolysis rate less than 5% [52]. Thus, stable immobilization of drugs, including synthetic analogs of antimicrobial peptides, onto a nanomaterial could be a method to overcome their limitations and side effects. Considering recent data that indicate some limitation for ceragenin use in systemic application [28], the antibacterial effect of MNP-CSA-13 was assessed against luminescence of *P. aeruginosa* Xen 5 in PBS containing 50% of different body fluids (Figure 7B). A significant decrease of bacterial luminescence was observed after addition of CSA-13, MNP and MNP@CSA-13 to cerebrospinal fluid, abdominal fluid and blood plasma specimens. Moreover, we observed that MNP-CSA-13 activity was higher than activity of CSA-13. It was also noted that free and functionalized CSA-13 possess the ability to bind bacterial membranes. Rapid interaction between *P. aeruginosa* cells and CSA-13 labeled with a fluorescent probe - BODIPY-succinimidyl ester (CSA-119) was recently reported. This study underlines the high affinity that positively charged ceragenins have for negatively charged bacterial membranes [39]. In agreement with reported results, we observed that ceragenin functionalized MNPs possess positive zeta potential (+39 eV) promoting the interaction with bacterial membrane surface characterized by negative zeta potential (−15 eV). Additionally after MNP@CSA-13 addition to bacteria suspension, a change of zeta potential from −15 eV to −6 eV was observed. In agreement with our results, Thill *et al.* revealed that cationic charged nanoparticles interact with negatively charged bacterial membranes via electrostatic interaction [53].

**Figure 8:**
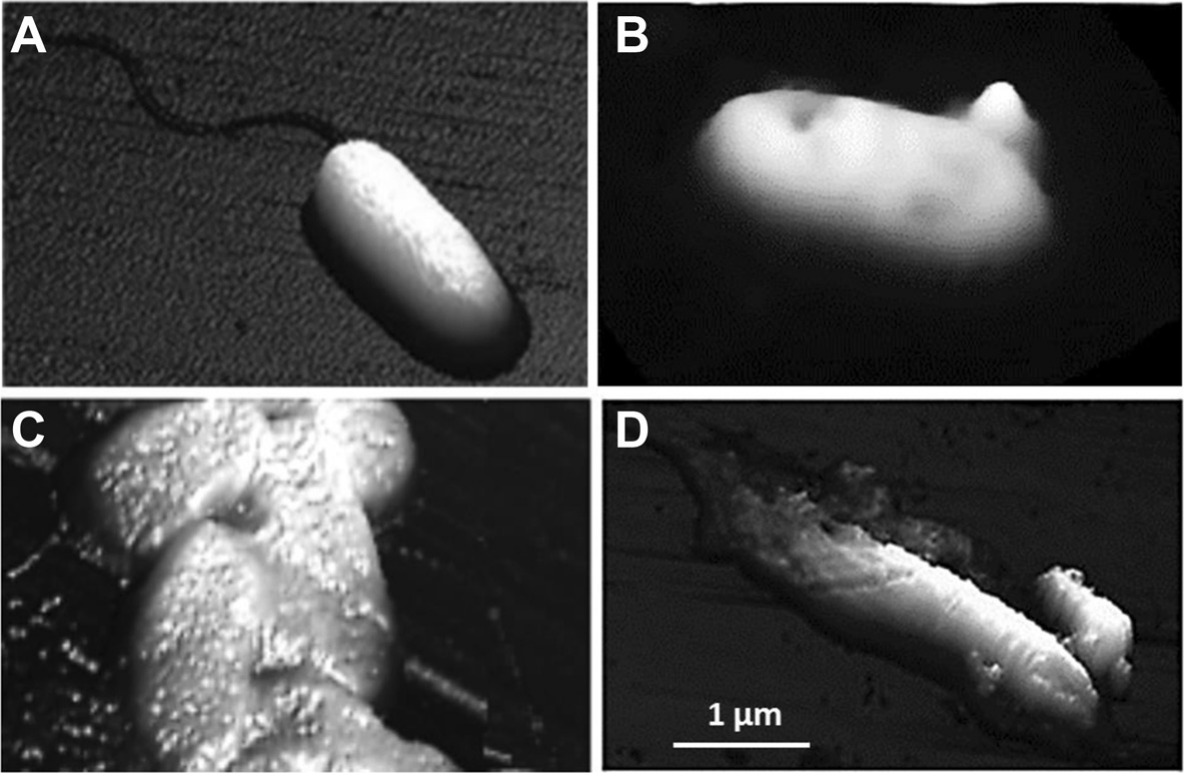
*P. aeruginosa* cell disruption under MNP-CSA-13 treatment, evaluated using atomic force microscopy. Images recorded before **(A)** and after CSA-13 **(B)**, MNP **(C)** and MNP-CSA-13 **(D)** addition. Analysis of cell surface topography showed increasing membrane roughness and leakage of the intracellular contents.

To confirm the antibacterial activity of ceragenin functionalized magnetic nanoparticles we utilized atomic force microscopy (AFM) to identify changes in cell morphology and mechanical properties of *P. aeruginosa* upon MNP-CSA-13 treatment (Figure 8). The results indicate that MNP-CSA-13 adhered to bacteria and then disrupted the cell membrane with leakage of the intracellular contents. Addition of unfunctionalized MNPs changes the bacterial shape and induce bacteria aggregation. Similarly to our finding De *et al.* revealed increases in membrane premeability of *P. aeruginosa* cells after treatment with lanthanium calcium manganate nanoparticles [54]. Disorganization of Gram-negative bacteria membranes was also confirmed for zinc oxide nanoparticles [55]. Additionally, our previous report indicates that magnetic nanoparticles possess the ability to aggregate *P. aeruginosa* cells due to interaction with their cell-wall component [25]. Combining CSA-13 antibacterial potential with the magnetic properties of MNP may have prospects for *in vivo* applications as a contrast in magnetic resonance imagining and novel tool to monitor infection sites in real-time. In a recent study the use of MNP in magnetic resonance imaging was explored [2], and compared to the MNP in the study the nanosystem presented in this work shows higher stability due to covalent CSA immobilization and possibility of controlled release of CSA-13, depending on the environmental pH [2]. However, the potential as a contrast agent needs to be explored in further studies.

## Conclusion

Presented results suggest a promising future for the use of nanoparticles functionalized by antimicrobial cationic lipids in biomedical applications as drug carriers in a controlled drug delivery system. The MNP-CSA-13 nanoparticles demonstrate dual benefits: a decrease of ceragenin hemolytic activity and increase of antimicrobial properties in body fluids. Furthermore, these functionalized MNPs demonstrate the utility of a platform for creating a multi-task nanosystem, which can be useful in theranostic applications.

## Methods

### Synthesis of magnetic nanoparticles functionalized by CSA-13 (MNP-CSA-13)

Iron oxide magnetic nanoparticles (MNP) were synthesized via modification of Massart’s methods [16]. The shell around the magnetic core was obtained via polycondensation of 3-aminopropylotrimethoxy silane, then the nanoparticles were suspended in ethanol and glutaraldehyde was added. After 60 minutes of stirring at room temperature, the precipitate was isolated by magnetic decantation. The collected precipitate was washed with ethanol. In the final step, MNPs functionalized by glutaraldehyde were re-suspended in ethanol containing CSA-13 and sonicated for 1 h. The precipitate was isolated by an external magnetic field, then washed three times with ethanol, three times with PBS and dried to powder at 60°C.

### Nanoparticle characterization

FT-IR spectra were recorded using a Thermo Scientific Nicolet 6700 FT-IR spectrophotometer. A thin layer of sample was placed in direct contact with an infrared attenuated total reflection (ATR) diamond crystal. All FT-IR spectra were collected in the wavenumber range of 4000 to 500 cm^−1^ by co-adding 32 scans with a resolution of 4 cm^−1^. Differential scanning calorimetry (DSC) was performed on a Mettler Toledo Star DSC system. Nitrogen was used as a purge gas (10 ml·min^−1^). Samples between 2 and 5 mg were placed in aluminum pans and heated from 25 to 450°C with a heating rate of 20°C/min. Thermogravimetric analysis (TGA) was recorded using a Mettler Toledo Star TGA/DSC unit. Nanoparticles (1–2 mg) were placed in the aluminum pans (40 μl) and heated from 50 to 800°C with a heating rate of 10°C/min. The morphology of MNPs was studied with transmission electron microscopy TEM/EDX (Tecnai G2 X-TWIN). A drop of the nanoparticle dispersion in THF was deposited onto a carbon-coated copper grid and dried in a vacuum oven at temperature 50°C.

### Quantitative analysis of CSA-13′s presence on MNP surface

As shown previously, the total number of amine groups present at the MNP surface in the MNPs@NH_2_ sample, determined by acid–base titration, was 0.42 mmol g^−1^ [56]. The total amount of CSA-13 on the MNPs was estimated based on weight loss during TGA analysis.

### Release of ceragenin from MNP-CSA-13

In order to determine the percentage of CSA-13 release in an *in vitro* setting, 10 μl of MNP-CSA-13 nanoparticles (10 mg/ml) were suspended into 10 ml of PBS (140 mM NaCl, 7.5 mM Na_2_HPO_4_; pH ~ 7.4) at pH = 7.4 and 5 respectively. In both environments, the cumulative amount of ceragenin released into the solution was measured by surface tension at different time intervals using the du Noűy ring method [57]. The surface tension was calculated from the radius of the ring and the tear-off force [57].

### Measure of the zeta potential of *Pseudomonas aeruginosa* cells and MNP-CSA-13

The zeta potential measurements on *P. aeruginosa* were taken at 25°C on a Zetasizer Nano ZS (Malvern Instruments, UK) apparatus, which is based on electrophoretic light scattering. Attenuation selection, voltage selection and measurement duration were set as automatic. The quality of the measurements was evaluated by inspection of the phase plot. Overnight cultures of bacteria were adjusted to 0.1 absorbance (600 nm) in PBS buffer (pH = 7.0). MNP-CSA-13 was dispersed in PBS buffer at concentration 2.5 mg/ml. The assay was performed three times.

### Antimicrobial testing

To compare the bactericidal activities of CSA-13 (0.7 μg/ml) and MNP-CSA-13 (5 μg/ml) and MNPs themselves (4.3 μg/ml) against *P. aeruginosa* PAO14, killing assay/CFU counting method was performed [58]. The concentrations of tested agents were calculated to added equal amount of free CSA-13 and CSA-13 molecules attached to the MNP surface. Briefly, *P. aeruginosa* was grown to mid-log phase at 37°C, re-suspended in PBS, and brought to 10^8^ CFU/ml (with the assumption that an optical density at 600 nm of 0.35 corresponds to 10^8^ CFU/ml). They were then diluted in PBS containing different concentrations of CSA-13, MNPs or MNP-CSA-13. After 1 h of incubation at 37°C the plates were transferred to ice and suspensions were diluted 10- to 1000-fold in PBS. Then, 10 μl aliquots were spotted on cetrimide-containing agar plates for overnight culture at 37°C, then CFUs were determined. In other set of experiment changes of chemiluminescence intensity of P. aeruginosa Xen5 after treatment with COL – colistin, CSA-13, MNP and MNP-CSA-13 measurements were determined as an additional method to assess bacteria viability. Chemiluminescence was evaluated using Labsystems Varioscan Flash (Thermo Scientific).

### MNP-CSA-13 activity against bacteria biofilm formation

A biofilm of *P. aeruginosa* was grown for 48 h at 37°C with and without the antibacterial agents. Each well was washed four times with deionized water to remove planktonic bacteria. Biofilm mass was evaluated using crystal violet (CV) staining (0.1%). Excess stain was rinsed off with deionized water and plates were dried. Then, 100 μl ethanol was added and optical density (OD) was determined at a wavelength of 570 nm. These OD values were considered as an index of bacteria adhering to the surface and forming a biofilm.

### Hemolytic activity

MNPs, CSA-13 and MNP-CSA-13 hemolytic activity was tested using human red blood cells (RBCs) suspended in phosphate-buffered saline (PBS) (hematocrit *~*5%) with a concentration of tested antibacterial agents ranging from 0–100 μg/ml. RBCs were incubated with tested agents for 1 h at 37°C. Relative hemoglobin concentration in supernatants after centrifugation at 2500 *g* was monitored by measuring optical absorbance at 540 nm. 100% hemolysis was taken from samples in which 1% Triton X-100 was added to disrupt all cell membrane.

### Antimicrobial activity of MNP-CSA-13 in different body fluids

To assess the ability of MNP, MNP-CSA-13 and free CSA-13 to kill bacteria in different compartments of the human body, changes of *P. aeruginosa* Xen 5 chemiluminescence (~ 10^9^ CFU/ml) in PBS mixed with 50% human blood plasma, serum, cerebrospinal fluid, peritoneal fluid, saliva and urine were assessed [28]. The luminomeric curves were recorded during 30 minutes after its addition.

### AFM imagining

AFM images were taken using an AFM BioScope Catalyst (LABSOFT, Bruker Nano Surfaces Division, Santa Barbara, USA). All measurements were performed in air-dried samples. The data was processed using NanoScope® AFM software.

## Abbreviations

AFM: Atomic force microscopy
AMPs: Antimicrobial peptides
APTMS: 3-aminopropylotrimethoxy silane
CAPs: Cationic antibacterial peptides
CSA: Ceragenins
CV: Crystal violet
DDS: Drug delivery systems
DSC: Differential scanning calorimetry
GDI: Glutaraldehyde
MNP@NH = CH(CH_2_)_3_CHO: Glutaraldehyde functionalized magnetic nanoparticles
MNP@NH_2_: Magnetic nanoparticles coated by aminosilane
MNP-CSA-13: Ceragenins functionalized magnetic nanoparticles
MNPs: Magnetic nanoparticles
TEM: Transmission electron microscopy
TGA: Thermogravimetric analysis
